# Neuropeptide Y Promotes Human M2 Macrophage Polarization and Enhances p62/SQSTM1-Dependent Autophagy and NRF2 Activation

**DOI:** 10.3390/ijms232113009

**Published:** 2022-10-27

**Authors:** Elisabetta Profumo, Elisa Maggi, Marzia Arese, Claudio Di Cristofano, Bruno Salvati, Luciano Saso, Rita Businaro, Brigitta Buttari

**Affiliations:** 1 Department of Cardiovascular and Endocrine-Metabolic Diseases, and Aging, Italian National Institute of Health, 00161 Rome, Italy; 2 Department of Medico-Surgical Sciences and Biotechnologies, Sapienza University of Rome, 04100 Latina, Italy; 3 Department of Biochemical Sciences “A. Rossi Fanelli”, Sapienza University of Rome, 00185 Rome, Italy; 4 Department of Surgical Sciences, Sapienza University of Rome, 00161 Rome, Italy; 5 Department of Physiology and Pharmacology Vittorio Erspamer, Sapienza University of Rome, 00185 Rome, Italy

**Keywords:** neuropeptide Y, macrophages, cytokines, autophagy, NRF2, atherosclerosis

## Abstract

Neuropeptide Y (NPY) is an abundantly expressed peptide capable of modulating innate and adaptive immune responses and regulating chemotaxis and cytokine secretion by macrophages. Abnormal regulation of NPY is involved in the development of atherosclerosis. The inflammatory infiltrate within atherosclerotic plaque is characterized by accumulation of macrophages, which are subject to reprogram their phenotypes in response to environmental signals. Macrophage number and phenotype influence plaque fate. Here, we investigated the effect of NPY on the changes in phenotype and functions of human macrophages, from the pro-inflammatory phenotype M1 to the reparative M2, indicative of atherosclerosis regression or stabilization. Human monocytes were differentiated in vitro into macrophages with M-CSF (M0) and polarized towards an M1 phenotype with IFN-γ plus LPS M(IFN-γ/LPS) or M2 with IL-10 (M IL-10) and further challenged with NPY (10^−7^–10^−9^ M) for 8–36 h. Cell phenotype and functions were analyzed by immunofluorescence and immunochemical analyses. NPY affected macrophage surface markers and secretome profile expression, thus shifting macrophages toward an M2-like phenotype. NPY also prevented the impairment of endocytosis triggered by the oxysterol 7-keto-cholesterol (7KC) and prevented 7KC-induced foam cell formation by reducing the lipid droplet accumulation in M0 macrophages. NPY-treated M0 macrophages enhanced the autophagosome formation by upregulating the cell content of the autophagy markers LC3-II and p62-SQSTM1, increased activation of the anti-oxidative transcription factor NRF2 (NF-E2-related factor 2), and subsequently induced its target gene *HMOX1* that encodes heme oxygenase-1. Our findings indicate that NPY has a cytoprotective effect with respect to the progression of the inflammatory pathway, both enhancing p62/SQSTM1-dependent autophagy and the NRF2–antioxidant signaling pathway in macrophages. NPY signaling may have a crucial role in tissue homeostasis in host inflammatory responses through the regulation of macrophage balance and functions within atherosclerosis.

## 1. Introduction

Atherosclerosis is a chronic inflammatory condition characterized by accumulation of pro-inflammatory macrophages and foam cells in the intima of the arterial wall. Foam cells appear when a dysregulation of lipid metabolism in macrophages occurs, and lipids accumulate within the cells. Of note, this process impairs macrophage immune functions [1]. Pro-inflammatory macrophages and foam cells both contribute to the plaque destabilization and rupture by secreting pro-inflammatory cytokines and matrix metalloproteases [2]. A simplified view shows that M1 macrophages promote plaque inflammation by secreting pro-inflammatory cytokines, whereas M2 macrophages resolve plaque inflammation by secreting anti-inflammatory cytokines and by stimulating angiogenesis and phagocytosis [3,4]. Macrophages are extremely plastic cells, which quickly react in response to injury, infection, and other types of noxious conditions such as hypoxia and metabolic stress by switching their functional phenotype to fulfil a pivotal role in host defense, wound healing, and immune regulation [5,6,7].

Several factors have been implicated in mediating M1 and M2 polarization [8,9,10]. In the plaque microenvironment M1/M2 balance may be influenced by growth factors, cytokines, and several other factors such as oxidized LDL and hemoglobin [11]. Of note, M1 and M2 activation phenotypes represent two ends of a functional spectrum of macrophage polarization state [12]. More recent experimental findings led to a substantial update of monocyte–macrophage nomenclature to include the nature of the polarizing signal; Murray and colleagues have proposed researchers describe stimulation scenarios and adopt a nomenclature linked to the activation standards, i.e., M(IL-4), M(Ig), M(IL-10), M(GC), M(IFN-γ), M(LPS) [13].

However, limited information exists on nervous-system-derived signals and processes governing the tuning of M1/M2 macrophage balance. Clinical and experimental evidence demonstrating bidirectional communications between the nervous system and the immune system has been accumulated [14]. Experimental in vivo and in vitro studies have demonstrated that regulatory neuropeptides, which act as neurotransmitters and neuromodulators, also have pro- and anti-inflammatory activities, thus attracting attention as therapeutic targets for inflammatory diseases. A recent study demonstrated that the neuropeptide galanin is expressed in human immune cells, particularly macrophages [15]; it acts as a potent modulator of cytokine and chemokine expression, thus playing a role in the fine-tuning of the macrophage-driven immune response. Exposure to chronic psychological stress is a risk factor for many diseases, including cardiovascular diseases (CVD). Psychological stress causes dysregulation of the sympathetic nervous system (SNS) and hypothalamic–pituitary adrenal (HPA) axis, which collectively promote inflammation, atherosclerosis, and subsequent CVD risks. Chronic and acute stress increase plasma adrenaline, noradrenaline, and neuropeptide Y (NPY) levels [16]. The latter consists of a 36 amino acid linear peptide recognized by NPY receptors belonging to the A G-protein-coupled receptor (NPY Y1, Y2, Y4, and Y5) class [17]. NPY has a critical role in the maintenance of homeostasis in the immune system and in coping with stress conditions. As a major mediator of the stress system, NPY shows pro-inflammatory and anti-inflammatory actions depending on the interaction with different Y receptor subtypes in relation to the cell type, age, and animal strain [18,19,20,21,22]. NPY has been shown to inactivate natural killer (NK) cells and macrophages, to inhibit expression of pro-inflammatory cytokines, and to promote anti-inflammatory cytokines such as transforming growth factor (TGF)-β1/interleukin (IL)-10 [23,24]. Recently, we showed that NPY may have an opposing effect on dendritic cells, since on the one hand, it induces migration of human dendritic cells via the Y1 receptor, but on the other hand, it exerts anti-inflammatory actions by inducing T helper 2 (Th2) polarization [25]. However, information regarding NPY effects on macrophage biology is lacking, especially on the regulation of macrophage balance and functions.

The role of NPY in the onset and course of atherosclerosis is still largely debated [26]. It has been demonstrated that unstable atherosclerotic plaques show a higher expression of NPY than stable plaques and that the induction of NPY in and around atherosclerotic lesions might enhance neoangiogenesis and promote lesion vulnerability. In several animal and in vitro studies, it has been shown that NPY exerts pro-atherogenic actions during stress, vascular injury, and ischemia [27], even though contradictory results exist on the role played by the NPY-Y1R axis on atherosclerosis progression [28]. It is likely that a persistent high level of exogenous NPY is not beneficial due to its vasoconstrictive effect, even though this effect was observed only in patients with microvascular angina, but not in control subjects or coronary artery disease patients [29]. Recently, Qin et al. reported cardioprotective effects of NPY. The authors observed that NPY KO mice exhibited a more severe form of acute myocardial infarction (AMI) when compared to WT mice, as they showed a worse cardiac dysfunction, progressive cardiac inflammation and fibrosis, excessive apoptosis, and impaired angiogenesis. They also observed that the administration of exogenous NPY reversed this worse outcome, and also attenuated AMI in WT mice [30]. However, NPY may play different roles, protective or pathogenic, under various clinical and pathological conditions, so there are still molecular mechanisms that need to be investigated.

Recently, it has been shown that NPY can modulate autophagy in hypothalamic neurons [31]. Autophagy is a bulk protein degradation process that functions as a cellular quality control system to remove damaged proteins or organelles [32], and it also regulates inflammasome [33] and innate immune responses [34,35]. Indeed, recent data showed that autophagy is required to suppress M1 and promote M2 polarization in macrophages [36]. The induction of autophagy in macrophages may have a plaque-stabilizing effect.

In the present study, we investigated by immunofluorescence and immunochemical analyses whether NPY was able to modulate in vitro macrophage phenotype and functions and the mechanisms underlying these processes. For this purpose, human monocytes were differentiated in vitro into macrophages (M0) and then polarized towards an M1 phenotype with IFN-γ plus LPS (M(IFN-γ/LPS)), or M2 phenotype with IL-10 (M(IL-10)) and challenged with NPY. Our findings indicate that NPY has a cytoprotective effect on pro-inflammatory signaling by efficiently tuning M1/ M2 macrophage balance towards an anti-inflammatory profile and by enhancing p62/SQSTM1-dependent autophagy and NRF2 activation in M0 macrophages.

## 2. Results

### 2.1. NPY Skews M(IFN-γ/LPS) Macrophages towards Anti-Inflammatory Phenotype

**Figure 1:**
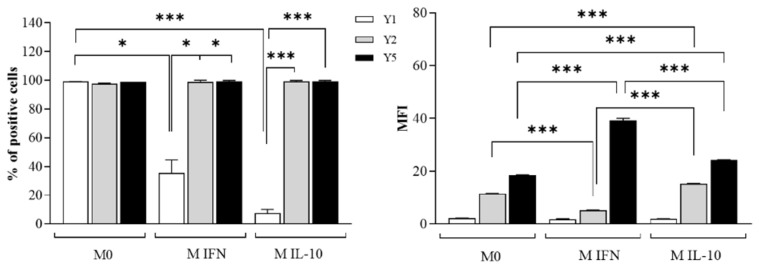
Flow cytometric analysis of neuropeptide Y receptors on untreated control M0 and polarized M(IFN-γ/LPS) and M(IL-10) macrophages. Cells were stained with primary antibodies anti-NPY Receptor Type 1 (NPY R Y1), type 2 (NPY R Y2), and type 5 (NPY R Y5), further detected by secondary antibodies conjugated to Alexa Fluor-488 and analyzed by flow cytometer. Histograms show the percentages of positive cells (%) and the mean fluorescence intensity (MFI). Results are expressed as mean value ± SD of 4 independent experiments. Significance was determined by one-way ANOVA followed by Tukey’s post hoc analysis; *: *p* < 0.05, ***: *p* < 0.001.

Flow cytometric analysis shows that all macrophage subsets express NPY R Y1, Y2, and Y5 (Figure 1).

When compared with M0, a low percentage of polarized M(IFN-γ/LPS) and M(IL-10) macrophages resulted positive for the NPYR Y1 receptor, even though NPYR Y1 expression (MFI) does not differ between the macrophage subsets. After polarization with IFN-γ and LPS, M(IFN-γ/LPS) macrophages downregulate the NPYR Y2 expression and upregulate NPYR Y5 (MFI), whereas M(IL-10) macrophages induced to polarize with IL-10 to upregulate the expression of both NPY receptors. Of note, M(IFN-γ/LPS) macrophages express the highest NPYR Y5 levels among macrophage subsets.

**Figure 2:**
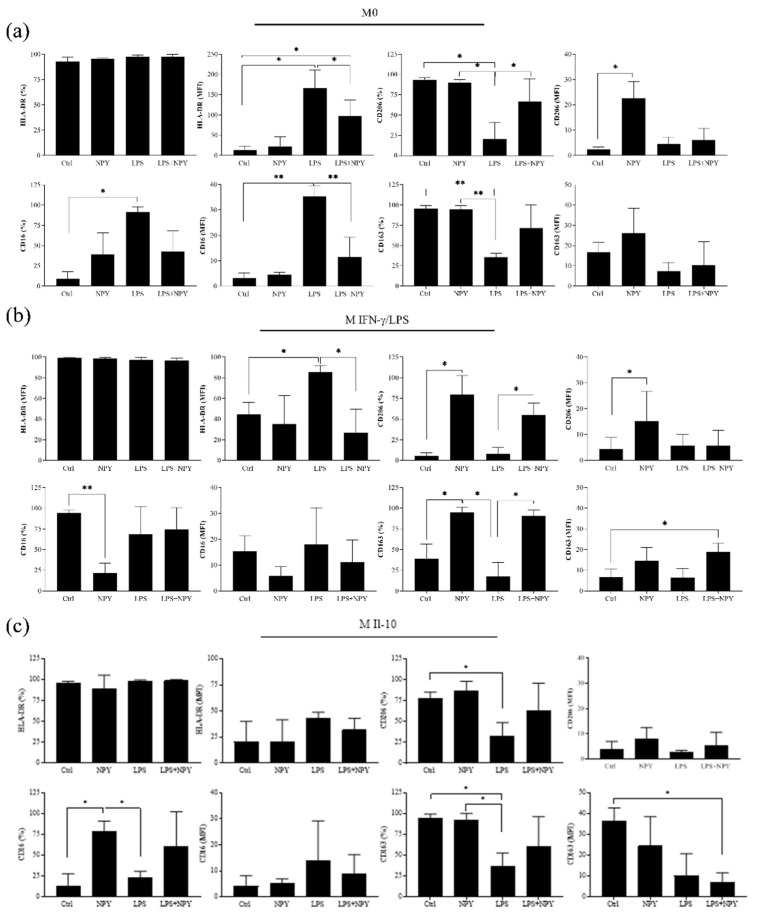
Flow cytometric analysis of the M1 markers HLA-DR and CD16 and the M2 markers CD206 and CD163 on (**a**) M0, (**b**) polarized M(IFN-γ/LPS), and (**c**) polarized M(IL-10) macrophages. Macrophages (7 × 10^5^ cells per mL) were stimulated or not with 10^−8^ M NPY in the presence or absence of LPS in complete medium for 24 h and then analyzed for surface marker expressions by flow cytometry. Histograms show the percentages of positive cells (%) and the mean fluorescence intensity (MFI). Results are expressed as mean value ± SD of 4 independent experiments. Significance was determined by one-way ANOVA followed by Tukey’s post hoc analysis; *: *p* < 0.05, **: *p* < 0.01.

Dose–response experiments demonstrated that 10^−7^ M was the highest tolerated concentration of NPY that did not affect macrophage viability in the trypan blue exclusion assay and cell morphology (% of cell viability in M0: Ctrl vs. 10^−6^ M NPY: 93.5 ± 2.8, 66.6 ± 3.5, *p* ≤ 0.001; Ctrl vs. 10^−7^ M NPY: 93.5 ± 2.8 vs. 89.3 ± 4.7, n.s. *p* > 0.05, *N* = 3, one-way ANOVA followed by Tukey’s post hoc analysis). We selected 10^−8^ M of NPY to investigate the surface antigen expressions and cytokine macrophage secretory capacity. The flow cytometric analysis of the M1- and M2-related surface antigens HLA-DR, CD16, CD206, and CD163 was conducted in M0, M(IFN-γ/LPS), and M(IL-10) macrophages (Figure 2a–c).

The majority of macrophages express both the M1 marker HLA-DR and CD16 and the M2 marker CD206 and CD163 (Figure 2). The treatment with NPY alone significantly increased CD206 on M0 (MFI) and M(IFN-γ/LPS) (% and MFI) and CD163 (%) on M(IFN-γ/LPS), whereas it downregulated CD16 (%) on M(IFN-γ/LPS) (Figure 2b,c). In the M(IL-10), NPY induced a significant increase in the percentage of CD16-positive cells of a higher order of magnitude than that observed with LPS (Figure 2c). As expected, LPS increased CD16 (% and MFI) and HLA-DR (MFI) on M0 and HLA-DR (MFI) on M(IFN-γ/LPS), and downregulated CD206 (%) and CD163 (%) on M0 and M(IL-10) macrophages. The treatment with NPY prevented the increase in HLA-DR expression and the downregulation of CD206 and CD163 expression mediated by LPS on M(IFN-γ/LPS). Analysis of surface antigen expressions of M0 macrophages shows a reduction in CD16 after the co-treatment with NPY and LPS.

**Figure 3:**
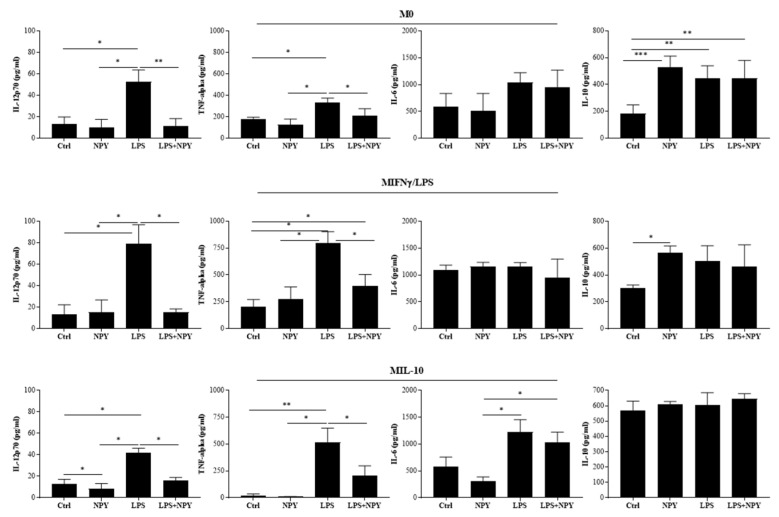
Secretome profile of cytokines in M0 and polarized M(IFN-γ/LPS) and M(IL-10) macrophages. Macrophages (7 × 10^5^ cells per mL) were stimulated or not with 10^−8^ M NPY in the presence or absence of LPS in complete medium. Supernatants were collected after 24 h to measure cytokines by specific ELISA experiments. Results are expressed as mean value ± SD of 4 independent experiments. Significance was determined by one-way ANOVA followed by Tukey’s post hoc analysis; *: *p* < 0.05, **: *p* < 0.01, ***: *p* < 0.001.

After incubation of the macrophages with LPS, we observed in the cell supernatants of all macrophage subsets a significant increase in the release of the pro-inflammatory cytokines IL-12 and TNF-alpha as compared with unstimulated cells (Figure 3), whereas the treatment of macrophages with NPY induced a significant increase in IL-10 in the M0 and M(IFN-γ/LPS) macrophages, and as expected it failed to impact IL-10 production significantly in IL-10-treated macrophages M(IL-10) (Figure 3).

The treatment with NPY prevented the increase in IL-12 and TNF-alpha in the cell supernatants of LPS-treated macrophages (Figure 3). These results suggest that NPY exerts an anti-inflammatory activity by switching macrophages toward an anti-inflammatory phenotype.

### 2.2. Neuropeptide Y Promotes Endocytosis in Macrophages

**Figure 4:**
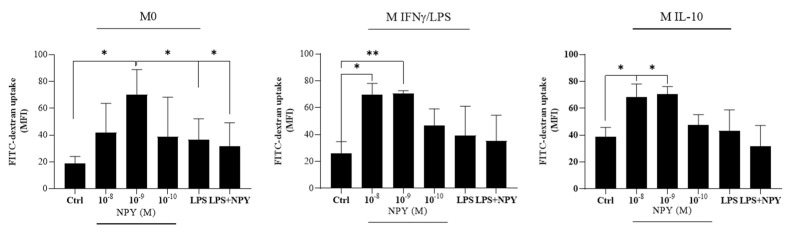
Flow cytometric analysis of endocytosis of FITC-dextran in M0 and polarized M(IFN-γ/LPS) and M(IL-10) macrophages stimulated with different concentrations (10^−8^–10^−10^ M) of NPY in the presence or absence of microbial LPS. After 24 h, treated and untreated cells (1 × 10^6^ cells/mL) were incubated with FITC-dextran (1 mg/mL) for 45 min. The cellular uptake was then analyzed by flow cytometry. Results are expressed as mean fluorescence intensity (MFI) (mean ± SD; *N* = 4). Significance was determined by one-way ANOVA followed by Tukey’s post hoc analysis; *: *p* < 0.05, **: *p* < 0.01.

Endocytosis is a crucial factor in macrophage-mediated host defense, which involves internalization and destruction of pathogens. In physiological conditions, macrophages contribute to maintaining tissue homeostasis through their activity of debris clearance, thus preventing excessive inflammation [37]. This activity is a hallmark of M2-like macrophages expressing higher levels of surface scavenger, mannose, and galactose-type receptors when compared to pro-inflammatory M1-like macrophages that show less endocytic ability [38]. Flow cytometric analysis showed that NPY-treated macrophage populations largely resulted positive for the FITC-dextran uptake. Of note, a bimodal effect of NPY on endocytosis of FITC-dextran was observed mainly in M0 macrophages. We found a significant decrease in the uptake of FITC-dextran when M0 macrophages were co-treated with the pro-inflammatory inducer factor LPS (Figure 4).

### 2.3. Neuropeptide Y Prevents 7-Keto-Cholesterol-Induced Foam Cell Formation in Macrophages

**Figure 5:**
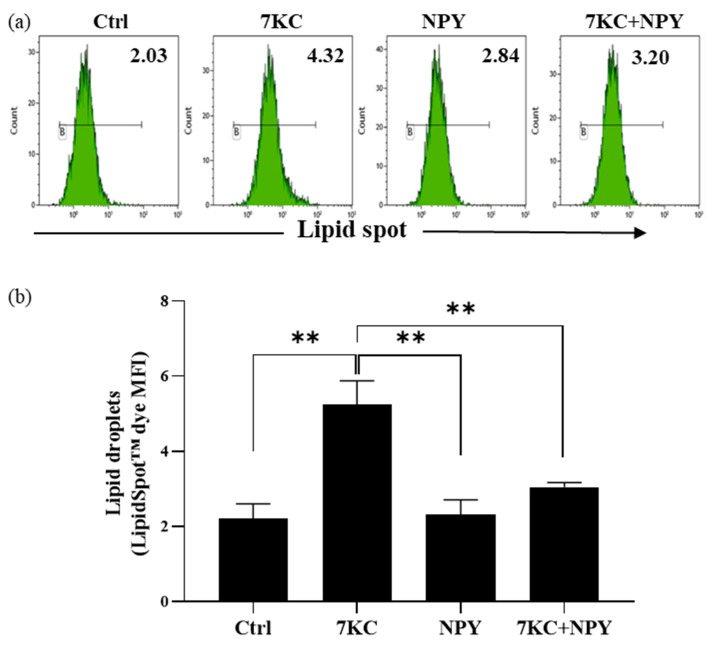
Determination of intracellular lipid droplet levels in M0 macrophages stimulated with 7-keto-cholesterol (7KC) in the presence or absence of NPY. M0 macrophages pretreated with 10^−8^ M NPY for 1 h were stimulated with 7KC for 24 h and then the median fluorescence intensity (MFI) of the intracellular LipidSpot™ dye was analyzed. (**a**) Representative flow cytometric analysis of intracellular lipid droplet levels MFI values is indicated. Histogram marker B designates the % of positive staining cells. (**b**) Flow cytometric analysis of lipid droplets (LipidSpot^TM^ MFI) expressed as mean ± SD from 4 independent experiments. Significance was determined by one-way ANOVA followed by Tukey’s post hoc analysis; **: *p* < 0.01.

To evaluate a possible role of NPY in the atherosclerotic process, we used an in vitro 7-oxysterol-induced foam cell formation model [39,40]. By flow cytometric analysis, we studied the ability of NPY to modulate the uptake of the LipidSpot™ dye induced by 7-keto-cholesterol (7KC) on M0 macrophages. As expected, the mean fluorescence intensity for the LipidSpot^TM^ significantly increased after 24 h exposure of M0 macrophages to 7KC (Figure 5). Of note, the 10^−8^ M NPY prevented 7KC-induced foam cell formation in M0 macrophages (Figure 5).

### 2.4. Neuropeptide Y Enhances Autophagosome Formation and p62 Accumulation in Macrophages

**Figure 6:**
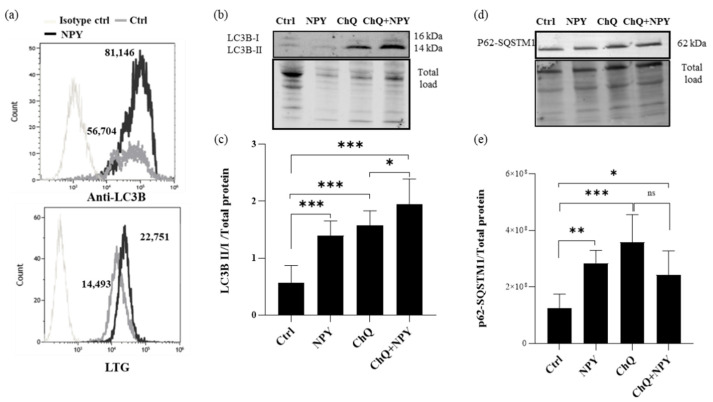
Flow cytometry and Western blotting analysis of autophagy markers in M0 macrophages treated with 10^−8^ M NPY for 24 h. (**a**) Representative flow cytometric analysis of autophagic marker LC3B and lysosomal dye LysoTracker (LTG) signals in M0 macrophages treated with 10^−8^ M NPY for 24 h. Cells were labeled with anti-LC3B-Alexa Fluor488 or isotype-Alexa Fluor488 or 10 µM LTG. Histogram overlays of LC3B or LTG expression of isotype control cells, untreated, and NPY-treated cells are shown. Median fluorescence intensity (MFI) values are indicated. (**b**–**e**) Western blotting analysis of LC3B and p62/SQSTM1 were performed in the whole-cell lysates of macrophages. Densitometric analysis of LC3BI/II panel (**c**) and p62/SQSTM1 (**e**) in M0 macrophages. Analyses were performed with ImageLab software (Biorad) and normalized to total protein. Data are expressed relative to the control total protein as mean ± SD of 6 independent experiments. Significance was determined by one-way ANOVA followed by Tukey’s post hoc analysis; *: *p* < 0.05, **: *p* < 0.01, ***: *p* < 0.001. Panel (**b**) and (**d**) show representative Western blotting results of LC3B and p62/SQSTM1, respectively.

Emerging studies have confirmed that autophagy is a critical regulatory mechanism of macrophage polarization in the progression of atherosclerosis [36,41]. By flow cytometric analysis, the upregulation of autophagic markers LC3B and lysosomal dye LysoTracker (LTG) signals were examined in M0 cells. Anti-LC3B labeling showed that untreated M0 cells had detectable amounts of LC3B (Figure 6a) compared to the isotype control and that cells treated with 10^−8^ M NPY for 24 h showed a 1.5-fold upregulation of LC3B above control levels (Figure 6a). Likewise, LTG showed a 1.5-fold upregulation of LTG signal (as MFI) when M0 cells were treated with NPY compared to untreated cells. Similarly, rapamycin treatment of M0 at 2 µM induced optimal LC3B upregulation at 24 h and a 1.6-fold LTG signal increase above control levels.

By using Western blotting analysis, we evaluated the overall status of autophagy in the macrophages by investigating the expression of the autophagic marker LC3-I/II, a widely used marker to monitor the autophagic process and p62/SQSTM1 on M0 macrophages treated with 10^−8^ M NPY or left untreated for 24 h. The lysosomal functional inhibitor chloroquine (ChQ; 50 µM) was used as a control of impaired autolysosomal degradation.

As shown in Figure 6b,c, the addition of NPY induced an increase in the transient autophagosomal membrane-bound form of LC3 (LC3-II) in M0 macrophages. In this condition, there was an increase in LC3-II induced by NPY in the presence of ChQ, and this increase was significantly higher than in cells treated with inhibitor alone, as shown in Figure 6b,c. P62, also known as SQSTM1/sequestome 1, serves as a link between LC3 and ubiquitinated substrates and is efficiently degraded by autophagy [42]. Since p62 accumulates when autophagy is inhibited and decreases when autophagy is induced, p62-SQSTM1 could be used as a marker to study autophagic flux. In our Western blotting experiments, at 24 h of exposure to NPY increased the p62 protein levels, similarly to ChQ, whereas NPY failed to further increase it in ChQ treated cells (Figure 6d,e), which suggests that the p62/SQSTM1 autophagic degradation pathway was slightly impaired by NPY.

**Figure 7:**
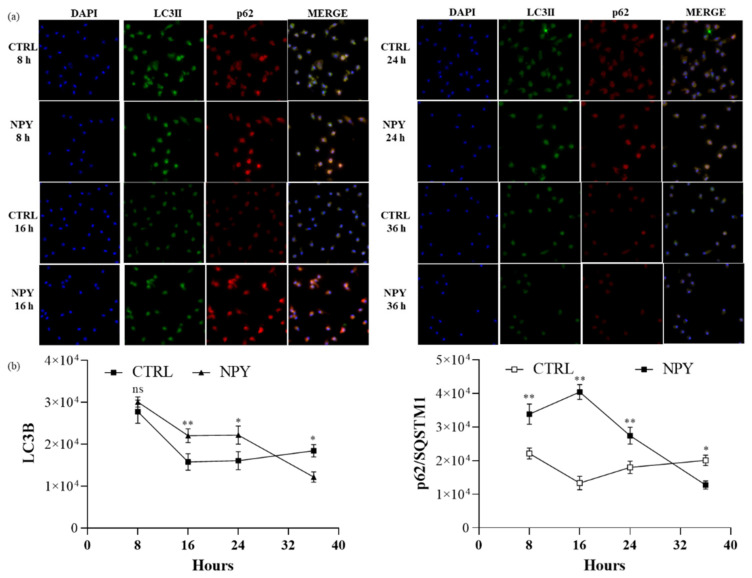
Immunofluorescent analysis of LC3B and p62/SQSTM1 expression in M0 macrophages at different time points after 10^−8^ M NPY exposure. (**a**) Representative immunofluorescent co-staining of LC3B and p62/SQSTM1. (**b**) Quantification of the intensity of the fluorescence signal for LC3B and p62 positive cells. DAPI was used to counterstain the nuclei. The upright microscope Nikon Eclipse Ni-U with 60× magnification was used to capture micrographs. Immunofluorescence analysis was performed using the ImageJ software. Data are presented as the mean ± SD for each group (*N* = 3). Significance was determined by one-way ANOVA followed by Tukey’s post hoc analysis; *: *p* < 0.05, **: *p* < 0.01.

To rule out that the autophagic flux was impaired by NPY in macrophages, the autophagy markers were analyzed by immunofluorescence microscope imaging at different time points after NPY exposure. The upregulation of LC3B reached a maximum at 8 h after NPY exposure, whereas p62/SQSTM1 reached a maximum at 16 h. Subsequently, the upregulation of autophagy markers gradually decreased over 36 h. The fluorescence analysis of LC3B and p62/SQSTM1 suggested similar patterns and were represented from three independent experiments. These data suggested that a time-dependent change in autophagy occurred in NPY-treated M0 cells and further supported the fact that it could activate autophagy in macrophages. Of note, autophagy markers remained high in untreated cells (Figure 7), thus confirming a basal autophagy in human primary macrophages.

### 2.5. Neuropeptide Y Promotes Human NRF2/HO-1 Activation in M0 Macrophages

**Figure 8:**
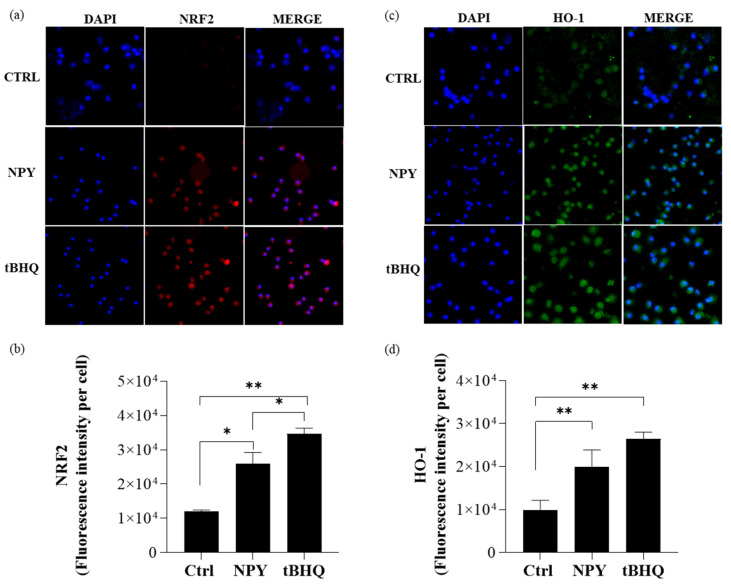
Immunofluorescent analysis of NRF-2 and HO-1 expression in M0 macrophages treated with 10^−8^ M NPY and 10 µM tertbutylhydroquinone (t-BHQ). (**a**) Representative immunofluorescent staining of NRF2 in M0 macrophages treated with NPY or tertbutylhydroquinone (t-BHQ) for 8 h. (**b**) Quantification of the intensity of the fluorescence signal for NRF2-positive cells. (**c**) Representative immunofluorescent staining of HO-1 in M0 macrophages treated with NPY or tertbutylhydroquinone (t-BHQ) for 24 h. (**d**) Quantification of the intensity of the fluorescence signal for HO-1-positive cells. Immunofluorescence analysis was performed using the ImageJ software. DAPI was used to counterstain the nuclei. Data are presented as the mean ± SD for each group (*N* = 3). For (**b**,**d**), significance was determined by one-way ANOVA followed by Tukey’s post hoc analysis; *: *p* < 0.05, **: *p* < 0.01. The upright microscope Nikon Eclipse Ni-U with 60× magnification was used to capture micrographs.

We next examined the activation of NRF2 in macrophages since it has been reported that the accumulation of p62 under conditions of impaired autophagy results in the non-canonical activation of the transcription factor NRF2 by p62. In consideration of the pivotal role carried out by NRF2 in controlling the expression of antioxidant genes that ultimately exert anti-inflammatory functions [43], we investigated the activation level of this transcription factor in macrophages exposed to NPY to evaluate the role of NRF2 in NPY-induced M2 polarization. The immunofluorescence images showed that at 8 h NPY, similarly to tertbutylhydroquinone (t-BHQ), a positive inducer of NRF2, triggered NRF2 activation, which was fully expressed in nuclei (Figure 8a,b). This upregulation of NRF2 levels was associated with an increase in expression of the NRF2 target gene *HMOX1* coding for heme oxygenase-1 (HO-1) at 24 h (Figure 8c,d).

These data indicate that NPY activates the NRF2/HO-1 signaling pathway in macrophages, thus playing an important role in the fine balance of macrophage functions.

## 3. Discussion

Our in vitro study provides new information showing the ability of NPY to exert a cytoprotective effect on human macrophages by preventing the expression of an inflammatory phenotype and stimulating anti-inflammatory functions, thus shifting human macrophages toward an anti-inflammatory M2-like phenotype. We also provide evidence that NPY effects on the M0 macrophage phenotype and functions are concomitant with the induction of autophagy and the activation of the p62/NRF2/HO-1 pathway. Our data demonstrate that polarization of macrophages in the presence of NPY provides the capacity to restrain the inflammatory response induced by bacterial LPS, and that simultaneously enhances expression of the regulatory cytokine IL-10.

Neuropeptide Y is an abundant sympathetic co-transmitter, widely found in the central and peripheral nervous systems and with diverse roles in multiple physiological and pathophysiological processes, such as cardiovascular diseases. In animal models and in humans, circulating NPY levels resulted elevated both in plasma and in tissues [44,45,46]. Our findings here suggest that even though a higher concentration of NPY (10 nM) than that reported in vivo was used to evoke effects on macrophage phenotype and functions, the treated cells maintained their viability, thus suggesting that this NPY concentration is likely to be physiologically relevant in vivo and mostly under various clinical and pathological conditions. The anti-inflammatory activity of NPY is supported by different findings. Surface marker phenotyping showed that NPY-stimulated M(IFN-γ/LPS) macrophages increased the expression of the M2 marker CD163 and CD206 scavenger receptors, suggesting an upregulation in the phagocytic clearance functions. Notably, the effect of NPY was less pronounced on these phagocytic receptors when it was added on M(IL-10) macrophages, even though NPY upregulated CD16, a low-affinity Fc receptor for IgG antibodies that is likely to positively influence the phagocytosis of antibody–antigen complexes, further increasing the anti-inflammatory clearance activity exerted by these cells. Further evidence of NPY anti-inflammatory effects on M(IFN-γ/LPS) macrophages is given by its ability to prevent the increased expression of the M1 activation marker HLA-DR in M(IFN-γ/LPS) macrophages mediated by LPS, suggesting a downregulation of macrophage function as antigen-presenting cells that reduce activation of adaptive immune responses. As expected, NPY increased the production of IL-10, a key anti-inflammatory and regulatory cytokine, thus confirming its ability to skew macrophages towards an M2-like anti-inflammatory phenotype. Our results on the ability of NPY to affect the M1 pro-inflammatory phenotype are consistent with findings by Qin et al., both in vivo and in vitro, showing a shift in the M1 pro-inflammatory macrophages to the M2 reparative phenotype induced by NPY during the cardiac repairing process via Y1R/p38/NF-κB signaling, which was blocked by deleting NPY or by pharmacologically inhibiting Y1R with its antagonist BIBP 3226 [30].

Our results demonstrating the ability of NPY to reduce LPS-induced production of the pro-inflammatory cytokines IL-12 and TNF-α are in line with the data reported by Ferreira et al., who observed that NPY, acting via the Y1R, inhibits LPS-induced microglial activation and reduces the associated release of IL-1β [47]. Our findings of NPY to counteract the pro-inflammatory signaling triggered in macrophages by LPS also agree with previous reports in animal models [48,49,50]. A possible mechanism for the anti-inflammatory activity of NPY has been proposed by Qin et al., pointing to its ability to interfere with the p38 MAPK and NF-κB- signaling to inhibit the pro-inflammatory response of macrophages [30].

Additional information on macrophage features were obtained from our investigation of macrophage endocytic activity, a feature directly related to macrophage functions. Our study showed that NPY enhances endocytosis functions in all three macrophage populations, showing a bimodal effect. The NPY is reported to possess bimodal features. Accordingly, its biological action has been described not only in a concentration-dependent fashion [51], but also associated to the relative affinity with its receptors in the microenvironment [52,53]. The NPY-enhanced endocytosis result is in line with the increased expression of the endocytic receptor CD206 observed in NPY-stimulated macrophages, thus indicating the activation toward an M2-like phenotype. In our experiments, NPY is able to prevent the loss of endocytosis function mediated by LPS in M(IFN-γ/LPS) macrophages. These data appear to be in contrast with a previous study of Ferreira and colleagues, who observed that NPY inhibits the Fc receptor-mediated phagocytosis in LPS-activated microglia [47] and IL-1β-induced microglial motility [54]. However, in our experiments of surface marker phenotyping, the low-affinity IgG Fc receptor CD16 resulted downregulated in the M(IFN-LPS) and upregulated in the M(IL-10) stimulated with NPY, thus confirming that NPY does not play a unique role and, depending on the cell type and its degree of activation as well as on the concentration and treatment duration, it may exert different effects [54]. Taken together, our data and the observations reported by Ferreira et al. unanimously sustain the key role played by NPY in modulating the functional activities of microglia and macrophages and the consequent release of mediators during inflammation, thus promoting an anti-inflammatory microenvironment.

In our study, the evaluation of NPY receptors demonstrated that macrophages, during polarization, are accompanied not only by global changes in cell proteome but also by a significant increase in receptor endocytosis. Indeed, type Y1 NPY R was downregulated in polarized M(IFN-γ/LPS) and M(IL-10) macrophages. Cells use endocytic downregulation as a means to turn the signal off. However, the internalized receptors may continue to signal in the cell interior [55], and it takes time to degrade internalized receptor–ligand complexes. Thus, further research on the interplay between the endocytic response and receptor signaling needs to be performed before starting NPY Y receptor drug discovery.

Previous studies showed that excess free cholesterol is stored as lipid droplets in macrophages and produces a foam cell morphology [56,57]. Since foam cell formation due to lipid droplet accumulation in macrophages is believed to play a crucial role in the progression of early atherosclerotic lesions and subsequent inflammation [58], we next evaluated the effects of NPY on 7KC-induced lipid droplets in M0 macrophages. Of note, due to the difficulty encountered in obtaining sufficient quantities of polarized monocyte-derived macrophages, the results relative to foam cell formation, autophagy, and NRF2/HO-1 pathways were performed only in M0 macrophages. Our results indicated that NPY reduces the accumulation of lipid droplets in macrophages treated with 7KC, thus further suggesting that the exposure of macrophages to NPY influences their polarization toward an anti-inflammatory phenotype through the influence on the lipid metabolism, modulating foam cell behavior and inhibiting lipid accumulation. Our results on the ability of NPY to reduce lipid uptake and foam cell formation differ with previous findings by Choi et al., who observed an increased lipid uptake induced by NPY in vascular smooth muscle cells (VSMC) from eNOS-deficient mice [59]. The differences in results coming from the two cell models are likely related to the heterogeneity and plasticity of these cells, which are highly specialized in sensing the microenvironment and modify their properties accordingly. Thus, it is not surprising and rather expected to find some different responses between VSMC and M0 macrophages. The discrepancies between our and Choi’s results could also be ascribed to the occurrence of eNOS deficiency, an enzyme that plays a critical suppressive role in the control of M1 macrophage activation [60].

Accumulating evidence shows that the dysfunctional autophagy plays a key role in atherosclerosis [61,62,63]. The impaired macrophage autophagy increases the immune response in obese mice by promoting pro-inflammatory M1 macrophage polarization [36] Our present data showed that NPY enhanced the autophagosome formation by upregulating the cell content of the autophagy marker LC3-II and p62-SQSTM1 and by increasing the transcription of the antioxidant factor NRF2 and the subsequent induction of its target gene, *HMOX1* that encodes HO-1. Analysis at 24 h suggests a slight impairment of the p62/SQSTM1 autophagic degradation pathway by NPY. It should be recognized that our observations in human macrophages are inconsistent with the previous report showing that NPY enhances autophagic flux by stimulating the p62 degradation pathway in hypothalamic neurons [31]. Although the p62 accumulates in NPY-treated macrophages, the effect is reversed quickly, so that p62 levels were decreased below that observed in the untreated control cells at 36 h, thus suggesting NPY to exert a stimulatory effect on autophagosome formation in macrophages. Further studies are needed to elucidate whether the increased p62 levels may be ascribed to NPY’s effects on other protein degradation systems, such as the ubiquitin–proteasome system [64], or its ability to transcriptionally upregulate p62.

In addition to its role in autophagy, p62 is involved in the antioxidant defense mechanism by activating the NRF2 transcription factor [65]. It has been shown that the accumulation of p62 results in its binding to Keap1, the E3 ligase responsible for the ubiquitination of NRF2. The binding of p62 to Keap1 suppresses NRF2 degradation by the UPS and causes NRF2 to move into the nucleus. Nuclear NRF2 induces a set of antioxidant enzymes that include HO-1 [66,67]. The activation of the NRF2/HO-1 pathway observed in this study might, therefore, represent a cellular cytoprotective mechanism through which NPY promotes anti-inflammatory mechanisms. Several reports have demonstrated a crucial role of NRF2 in tuning the balance of M1/M2 macrophages, thus identifying NRF2 as a molecular target to control macrophage inflammatory response [68,69]. Somehow, consistent with our observation, it is reported that the central nervous system stimulant methamphetamine induces the NRF2 signaling pathway through impaired autophagic flux and p62 accumulation in a mouse atrial cardiac cell line [70].

By regulating the expression of multiple genes involved in cholesterol influx and release, antioxidant enzymes, scavenger receptors, and ABC transporter proteins, NRF2 has been demonstrated anti-atherogenic effects in both in vitro and in vivo studies [71]. Nevertheless, some reports have uniformly concluded that the global loss of NRF2 results in the reduction in atherosclerotic lesion size in ApoE^−/−^ mice [72,73,74,75,76], but has different effects on plaque morphology depending on the plaque type [76]. Further investigations are needed in order to verify the significance of the NRF2 pathway associated with foam cells/fatty streak development before it is developed as a potentially novel therapeutic target.

Our study takes research into how NPY by interacting with its receptors exerts a cytoprotective effect on human macrophages a small step ahead. Overall, our findings, along with current knowledge, provide a critical link between autophagy dysregulation and prolonged NRF2 signaling in a p62-dependent manner and add some new information that could be useful to explain, at least in part, the close relationship of NPY and macrophages with the pathophysiological mechanisms underlying arteriosclerotic cardiovascular disease [26,27,28], as well as the contradictory results obtained in animal models of atherosclerosis deficient for NPY [30] or NRF2 [72,73,74,75,76].

## 4. Materials and Methods

### 4.1. In Vitro Cell Stimulation/Polarization

Peripheral blood mononuclear cells (PBMCs) were obtained from buffy coats of healthy blood donors. Monocytes were purified by incubating PBMCs with anti-CD14-coated microbeads (Miltenyi Biotec, Bergish Gladbach, Germany), followed by sorting with the magnetic device MiniMacs Separation Unit (Miltenyi Biotec, Bergish Gladbach, Germany), according to the manufacturer’s instructions [22]. To obtain monocyte-derived macrophages (termed M0 macrophages), adherent monocytes were cultured for 5–6 days in complete medium (RPMI 1640 supplemented with 1% nonessential amino acids, 1% sodium pyruvate, 50 U/mL penicillin, 50 g/mL streptomycin (Gibco, Karlsruhe, Germany), 5 × 10^−5^ M 2-mercaptoethanol (Merck, Darmstadt, Germany), and 10% fetal calf serum (Hyclone Laboratories, Logan, UT, USA) in the presence of 10 ng/mL recombinant human (rh) macrophage colony-stimulating factor (M-CSF). M0 macrophages were further polarized towards M1-like phenotype using 10 ng/mL rh interferon (IFN)-γ and 10 ng/mL toll-like receptor 4 (TLR4) ligand LPS (lipopolysaccharide from Escherichia coli strain 0111:B4, Sigma-Aldrich, St. Louis, MO, USA) (M IFN macrophages) or M2-like phenotype using 10 ng/mL rh interleukin (IL)-10 (M IL-10) in the presence or absence of human NPY (10^−11^ − 10^−7^ M; Bachem, Weil am Rhein, Germany) and/or LPS (0.2 μg/mL) for 8–36 h. All cytokines were purchased by Miltenyi Biotec. Macrophages were washed with warm phosphate-buffered saline (PBS) and harvested using TrypLE^TM^ Express Enzyme (Gibco, Karlsruhe, Germany). Cell viability was measured employing the trypan blue exclusion assay (Sigma-Aldrich) and cell morphology was checked by a light microscope (Nikon Eclipse Ni-U, Nikon Corporation, Tokyo, Japan).

### 4.2. Flow Cytometric Analysis of Macrophage Surface Markers and Endocytosis

To determine macrophage phenotypic surface markers, macrophages were stained with the following monoclonal antibodies (mAbs): phycoerythrin (PE)-CD163, fluorescein isothiocyanate (FITC)-CD206 and PE-Vio770-CD206 mAbs (Miltenyi Biotec), allophycocyanin (APC)-CD16 and APC-Alexa Fluor 750-HLA-DR (clone Immu357) mAbs (Beckman Coulter, Lane Cove, Australia), PE-CD1a and FITC-CD14 mAbs, or with isotype-matched control mAbs from PharMingen (PharMingen, San Diego, CA, USA) for 30 min at 4 °C. The receptor subtype surface expression of NPY on macrophages were assessed by staining with rabbit polyclonal primary antibodies anti-NPY Receptor Type 1 (NPY 1R), type 2 (NPY 2R), and type 5 (NPY 5R) (MyBioSource, Eersel, The Netherlands) and further detected by goat-anti-rabbit secondary antibodies conjugated to Alexa Fluor-488 (Thermo Fisher Scientific, Waltham, MA, USA). To determine mannose receptor-mediated endocytosis, macrophages (1 × 10^6^ cells/mL) were incubated with FITC-dextran (1 mg/mL; Sigma-Aldrich) for 45 min at 37 °C. Internalization ability was analyzed as the percentage and the mean fluorescence intensity (MFI) of FITC-positive cells as previously described [23]. In brief, dead cells were excluded by 1μM Sytox Blue nucleic acid staining (Molecular Probes, Carlsband, CA, USA). Cells were acquired by Gallios Flow cytometer, equipped with 3 lasers (405 nm, 488 nm, 633 nm, Beckman Coulter), and data were analyzed with Kaluza Analysis Software v. 2.1 (Beckman Coulter).

### 4.3. Secretome Profile of Cytokines in Macrophage Culture Supernatants

Supernatants from human macrophages (7 × 10^5^ cells per mL) treated or left untreated for 20 h in 24-well plates were collected, centrifuged, and stored at −80 °C. The levels of IL-12 p70, TNF-alpha, IL-6, and IL-10 into the conditioned medium were determined by enzyme-linked immunosorbent assay (ELISA; BD OptEIA™ Kits; BD Biosciences, San Diego, CA, USA) following the manufacturer’s instructions. The limits of detection were as follows: IL-12p70, 7.8 pg/mL; TNF-alpha and IL-10, 16 pg/mL; IL-6, 2.2 pg/mL.

### 4.4. Flow Cytometric Analysis of Intracellular Lipid Levels

In vitro model of foam cell formation induced by 7-oxysterols was previously described [39,40]. In brief, M0 macrophages (1 × 10^6^ cells/mL) were pretreated with NPY for 1 h and then exposed to 7KC (20 nm/L, Sigma-Aldrich) for a further 20 h in complete medium. Cells were stained with LipidSpot™ 488 Lipid Droplet Stains according to the manufacturer’s instructions (Biotium, Fremont, CA, USA). LipidSpot™ dyes are fluorogenic neutral lipid stains that rapidly accumulate in lipid droplets, where they become brightly fluorescent (Abs/Em: 427/585 nm). After 30 min of incubation in the dark at 37 °C, cells were centrifuged and the pellet was washed twice with ice-cold PBS/FCS and stained with DAPI (4 µg/mL, Thermo Fisher Scientific) to exclude dead cells. At least 5 × 10^3^ cells/sample were analyzed by flow cytometry (Gallios Flow Cytometer; Beckman Coulter).

### 4.5. Evaluation of Autophagy Markers and NRF2/HO-1

#### 4.5.1. Indirect Immunofluorescence Labeling

For immunofluorescence microscopy, cells were plated on chamber slides (Millicell EZ SLIDE 8-well glass from Millipore, Milan, Italy) at a density of 80 × 10^3^ cells/well, and were fixed after treatments with 4% paraformaldehyde (Sigma-Aldrich) at room temperature for 30 min. After washing with PBS, cells were permeabilized with 0.1% Triton-X100 (Sigma-Aldrich) in PBS for 5 min at room temperature. Cells were then washed with PBST, blocked with 0.1 M glycine in PBS for 20 min, and incubated with the primary antibodies: rabbit anti-LC3B (1:500; Novus Biologicals; Milan, Italy), mouse anti- p62-SQSTM1 (1:200; Abnova, Taipei, Taiwan, China), rabbit polyclonal anti-heme oxigenase 1 (1:500; Bioss antibodies, Beijing, China), and mouse anti-NRF2 (1:200; clone A-10, Santa Cruz Biotecnology, Heidelberg, Germany) in PBS at 37 °C for 90 min. After washing with PBST, fluorescence-labeled secondary antibodies were applied and incubated at 37 °C for 30 min: Alexa Fluor 488 goat anti-rabbit (Thermo Fisher Scientific) or Alexa Fluor 594 goat anti-mouse at a 1:100 dilution (Invitrogen, Carlsbad, CA, USA). Finally, the cells were marked with DAPI (Thermo Fisher Scientific) to highlight the nucleus and the glasses were mounted with aqueous mounting medium. Images were captured using an upright microscope (Nikon Eclipse Ni-U, 60× magnification, Nikon Corporation). Nikon NIS-Elements 5.0 imaging software (Nikon Corporation) was used for collecting images and for post-acquisition processing. The analysis was carried out in triplicate from three different fields.

Indirect intracellular labeling with rabbit polyclonal anti-LC3-II was carried out by incubation with fixed and permeabilized M0 macrophages. Briefly, 24 h after treatment with NPY or ChQ (Selleckchem, Verona, Italy), cells were fixed with BD FACS lysing solution and permeabilized with BD FACS permeabilizing solution (BD Biosciences) for 10 min each step. After that, cells were washed in PBS buffer stained with anti-LC3B polyclonal or rabbit immunoglobulin (as isotype control) (Novus Biologicals) for 2 h at room temperature. After this step, cells were washed in PBS and labeled with secondary fluorescent conjugate Alexa Flour 488 goat anti-rabbit IgG (Thermo Fisher Scientific) for 30 min. Cells were then washed and resuspended in PBS. The resulting cells were then analyzed by flow cytometer. Median fluorescent intensity (MFI) of LC3-II-AF-488 of the 520/30 nm signal of the cells was compared to determine relative increase in the LC3-II autophagic signal above resting control cells.

#### 4.5.2. LysoTracker Green Labeling

LysoTracker dyes (LTG, Thermo Fisher Scientific) label acidic spherical granules inside cells. To determine the autophagic flux, untreated and M0 treated for 24 h were loaded with LTG at a final concentration of 10 nM and incubated for 1 h at 37 °C. After incubation, cells were washed in PBS buffer and resuspended in 400 μL of PBS. The resulting cells were analyzed on a flow cytometer with excitation at 488 nm and LTG emission collected at 530/30 nm. LTG signals from samples were then compared by histogram analysis of MFI.

#### 4.5.3. Western Blot Analysis of Macrophage Lysates

Cells were removed rapidly and homogenized on ice with lysis buffer (CelLytic buffer, Sigma-Aldrich) plus protease and phosphatase inhibitors (protease inhibitor cocktail: 1 mM sodium fluoride, 1 mM sodium orthovanadate, and 1 mM sodium molybdate; 1 mM phenylmethylsulfonyl fluoride; and 1 mM phosphoinositidase C; all chemicals from Sigma-Aldrich)). Protein extracts were cleared by centrifugation and 30 μg protein was separated by electrophoresis in 4–15% Mini-PROTEAN^®^ TGX Stain-Free™ Precast Gel (Bio-Rad, Segrate, Italy). SDS-PAGE and transferred to Immobilon-P membranes (Millipore, Milan, Italy). After protein transfer, membranes were imaged for stain-free staining and total proteins were visualized by using ChemiDoc Imaging System (Bio-Rad) and quantified using Imagelab 6.0.1 (Bio-Rad) to correct for possible protein loading inaccuracy. The membranes were blocked with 5% (wt/vol) low-fat milk (Sigma-Aldrich) and 1% bovine-serum albumin (wt/vol; Sigma-Aldrich) in Tris-buffered saline (137 mM NaCl, 20 mM Tris·HCl, pH 7.6) containing 0.1% Tween 20 (Sigma-Aldrich) (TBS-T) for 1 h at room temperature. The membranes were incubated overnight at 4 °C with the primary antibodies rabbit anti-LC3B (1:500; Novus Biologicals), mouse anti-p62-SQSTM1 (1:200; Abnova). Appropriate peroxidase-conjugated secondary antibodies (1:10,000; Bio-Rad) were used to detect the proteins of interest by enhanced chemiluminescence (Clarity Western ECL Substrate; Bio-Rad) or fluorescence-conjugated secondary antibodies (1: 5000, goat anti-mouse starBright Blue 520 and goat-anti rabbit Dylight@680, Bio-Rad). Protein immunoreactive bands were visualized by chemifluorescence in a ChemiDoc Imaging System (Bio-Rad) and the optical density of the bands was quantified with the Imagelab 6.0.1 (Bio-Rad). The results were normalized to total protein and expressed as the relative amount compared with control or as relative intensity units. Some membranes were re-probed with a monoclonal anti-β-actin Ab (1:5000; Sigma-Aldrich) for equal protein loading control.

#### 4.5.4. Statistical Analysis

Mean values and standard deviations (SD) were calculated for each variable under study. The statistical analysis was performed by GraphPad Prism 8 software (San Diego, CA, USA). Normally distributed data were analyzed using one-way ANOVA with a Tukey post hoc test. Values of *p* < 0.05 were considered statistically significant.

## 5. Conclusions

In summary, our results demonstrate that NPY is able to tune M1/M2 macrophage balance towards an anti-inflammatory profile, and to promote the activation of the NRF2–antioxidant pathway and p62/SQSTM1-dependent autophagy. However, further studies are required to dissect the effects of NPY on p62-dependent NRF2 activation.

## Data Availability

Not applicable.
